# A New Overdispersed Integer-Valued Moving Average Model with Dependent Counting Series

**DOI:** 10.3390/e23060706

**Published:** 2021-06-02

**Authors:** Kaizhi Yu, Huiqiao Wang

**Affiliations:** School of Statistics, Southwestern University of Finance and Economics, Chengdu 611130, China; yukz@swufe.edu.cn

**Keywords:** integer-valued moving average model, counting series, dispersion test

## Abstract

A new integer-valued moving average model is introduced. The assumption of independent counting series in the model is relaxed to allow dependence between them, leading to the overdispersion in the model. Statistical properties were established for this new integer-valued moving average model with dependent counting series. The Yule–Walker method was applied to estimate the model parameters. The estimator’s performance was evaluated using simulations, and the overdispersion test of the INMA(1) process was applied to examine the dependence between counting series.

## 1. Introduction

Integer-valued time series can be encountered in numerous fields, such as epidemiology, insurance, and intraday stock transitions. The most widely used model is the integer-valued autoregressive (INAR) model, a recursive model first introduced by Alzaid and Al-Osh [1] and is similar to the traditional autoregressive (AR) model. Du and Li [2] generalized the model to the *p*-th order (which was called INAR(*p*) model) and proved the ergodic and Markov properties of the model. Similar to the continuous-valued moving average model, the *q*-th order integer-valued moving average model INMA(*q*) was introduced by Al-Osh and Alzaid [3], which is a slightly different form proposed by McKenzie [4].

Many researchers generalize the INAR model to deal with different real-life situations. Weiß [5] presented a new INAR(*p*) model showing possible marginal distributions of the DSD family. This model overcomes the difficulty of choosing the appropriate marginal distribution. Monteiro and Scotto [6] defined the periodic integer-valued autoregressive model, driven by a periodic sequence of independent Poisson-distributed random variables. Weiß [7] proposed the extended Poisson INAR(1) model, where the innovations are assumed to be serially dependent. Zhu [8] introduced a negative binomial INGARCH model to handle integer-valued time series with overdispersion and potential extreme observations. The study by Weiß [9] discussed threshold models for integer-valued time series with infinite range and briefly discussed new models for counting data time series with a finite range. Kang and Wang [10] generalized the mixture INAR(1) model based on mixing Pegram and binomial thinning operator. Li and Wang [11] proposed the first-order mixed integer-valued autoregressive process with zero-inflated generalized power series innovations, which contains the commonly used zero-inflated Poisson and geometric distributions. To handle the non-stationary integer-valued time series with a large dispersion, Kim and Park [12] introduced an integer-valued autoregressive process with a signed binomial thinning operator (INARS(*p*)).

Various modified thinning operators have been proposed to capture the specificity of real data, and many new INAR-type models have been defined. For example, Zheng and Basawa [13] introduced the random coefficient thinning operator while Ristić and Bakouch [14] proposed the negative binomial thinning operator. The *p*th-order integer-valued autoregressive process with signed generalized power series thinning operator was proposed by Zhang et al. [15].

The most significant generalization of the thinning operator was made by Ristić et al. [16] which they called the dependent Bernoulli thinning operator. They constructed the new sequence of the Bernoulli random variable allowing correlation between counting series. Based on this, Miletić Ilić et al. [17] proposed the new model, based on the mix of regular binomial thinning and dependent thinning operator. For more details of thinning operators, refer to Weiß [18].

MA-type models are very important in time series analysis. The method of moving average is generally popular in statistical and mathematical analyses. Some researchers study forecasting using the moving average method. Winters [19] analyzed the exponentially weighted moving average method for forecasting sales. Cox [20] proposed the weighted moving average method to predict the Markov series. Landauskas et al. [21] introduced an algebraic approach to select the appropriate weight coefficients for weight moving averages performing better than classical moving average predictors. Wind speed prediction using combined time series model and neural network prediction was studied by Nan et al. [22]. Other applied weight moving average methods in control charts. Alevizakos et al. [23] proposed the triple exponentially weighted moving average control chart (TEWMA), which improves the detection ability of the classical control chart. Capizzi and Masarotto [24] proposed the adaptive exponentially weighted moving average control chart (AEWMA), which weights past observations of the monitored process using a suitable function of the current error. Adegoke et al. [25] studied the multivariate homogeneously weighted moving average (MHWMA) control chart for monitoring a process mean vector.

Some researchers constructed MA-type models from the perspective of count time series. Brännäs and Quoreshi [26] used the INMA process to model the number of transactions for intraday stocks and extended the model to include explanatory variables. Brännäs and Hall [27] mainly focused on the estimation in the INMA model. The construction of the thinning operation from these studies is based on independent counting series, which is a strong assumption. Thus, to make the model more flexible in capturing the specificity of different data types, this assumption should be relaxed.

For small counts of intraday transactions in stocks per minute, the decision of buyer or seller could be affected by the public news, which means decisions from different individuals may not be uncorrelated. The change in inventories can sometimes be described as an INMA process. However, during a certain period, the change can be influenced by the same external factor. For the INMA model applying a discrete risk model, the number of claims for certain insurance will be affected by the same factor, such as natural disasters. Thus, these claims are no longer independent. The independence between counting series should be relaxed. Allowing the correlation between counting series is natural, and therefore the INMA model based on dependent counting series can be derived to handle different real data situations.

The rest paper is organized as follows. Section 2 presents the model construction and discussions on some relevant statistical properties, and Section 3 discussed the estimation of unknown parameters. Section 4 shows the numerical simulation results and give dispersion test for dependence between counting series, while the conclusions are given in Section 5.

## 2. The Model and Basic Properties

### 2.1. The Model Construction

The counting series ${\left\{U\right\}}_{i\in N}$ of the integer-valued model is defined as:

$$\begin{array}{c} U_{i}=\left(1-V_{i}\right)W_{i}+V_{i}Z \end{array}$$

${\left\{W\right\}}_{i\in N}$ is a sequence of i.i.d random variable with $W_{i}∼B\left(1,\beta \right)$, β∈[0,1]. ${\left\{V\right\}}_{i\in N}$ is a sequence of i.i.d random variable with $V_{i}∼B\left(1,\theta \right)$, θ∈[0,1]. *Z* is a random variable with Z∼B(1,β). The operator $\circ_{\theta }$ is defined by $\beta \circ_{\theta }\varepsilon_{t}=\sum_{i=1}^{\varepsilon_{t}}U_{i}$, it is the dependent Bernoulli thinning operator, where $\varepsilon_{t}$ is a non-negative integer-valued random variable. Based on this construction, we can easily verify that $E(U_{i})=\beta$, $Var\left(U_{i}\right)=\beta \left(1-\beta \right)$, $corr\left(U_{i},U_{j}\right)=\theta^{2}$, which has promising dependence between the counting series. Now we generalize the dependent count series to the INMA(*q*) process, For convenience, we use ∘ instead of $\circ_{\theta }$ to simplify the notation.

**Definition** **1** (Dependent Counting series Integer-valued Moving Average Model (DCINMA))**.** *The DCINMA(q) model is defined as:*

$$X_{t}=\varepsilon_{t}+\beta_{1}\circ \varepsilon_{t-1}+\ldots +\beta_{q}\circ \varepsilon_{t-q}$$

*$\beta_{j}\circ \varepsilon_{t-j},j=1,2,\ldots ,q$ is the dependent Bernoulli thinning operator, and the following conditions should be satisfied.*

*A1. ${\left\{\varepsilon_{t}\right\}}_{t\in N}$ is a sequence of i. i. d non-negative random variables.*

*A2. The counting variable ${\left\{U_{i}\right\}}_{i\in N}$ is independent of $\varepsilon_{t}$ for any i,t.*

*A3. $\beta_{j}\circ \varepsilon_{t-j}$, for any j=1,2,…,q are mutually independent.*

### 2.2. The Numerical Properties for DCINMA(q) Model

We denote $\mu_{\varepsilon }$ and $\sigma_{\varepsilon }^{2}$ as the mean and variance of term $\varepsilon_{t}$.

**Theorem** **1.** *The numerical characteristics of $\left\{X_{t}\right\}$ in Definition 1 are as follows:*

*(i)*  $E\left(X_{t}\right)=\mu_{\varepsilon }\left(1+\sum_{i=1}^{q}\beta_{i}\right)$
*(ii)*  $Var\left(X_{t}\right)=\sigma_{\varepsilon }^{2}+\sum_{i=1}^{q}\left[\mu_{\varepsilon }\beta_{i}+\mu_{\varepsilon }^{2}\theta^{2}\beta_{i}\left(1-\beta_{i}\right)-\mu_{\varepsilon }\left(\theta^{2}\beta_{i}-\theta^{2}\beta_{i}^{2}+\beta_{i}^{2}\right)+\beta_{i}^{2}\sigma_{\varepsilon }^{2}\right]$
*(iii)*  $$cov\left(X_{t},X_{t-k}\right)=\left\{\begin{array}{cc} \sigma_{\varepsilon }^{2}\sum_{i=0}^{q-k}\beta_{i}\beta_{i+k} & k=1,\ldots ,q\\ 0 & k\geq q+1 \end{array}\right.$$

**Proof.**  See Appendix A. ☐

**Theorem** **2.** *$\left\{X_{t}\right\}$ is the process defined in Definition 1, then $\left\{X_{t}\right\}$ is a covariance stationary process.*

**Proof.**  It can be seen from the Theorem 1 that the unconditional mean and unconditional variance of $X_{t}$ is a finite constant given the distribution of $\varepsilon_{t}$. Thus, $X_{t}$ is a stationary process. ☐

**Theorem** **3.** *$\left\{X_{t}\right\}$ is the process defined in Definition 1, then $\left\{X_{t}\right\}$ is ergodic in mean and autocovariance function.*

**Proof.**  See Appendix A. ☐

### 2.3. The Probability Generating Functions for DCINMA Model

Ristić et al. [16] derived the probability generating function of the $\sum_{i=1}^{n}U_{i}$ as follows:

$$\begin{array}{cc} \Phi_{U} & =E[s^{\left(U_{1}+U_{2}+\ldots +U_{n}\right)}]\\ & =\left(1-\beta \right){\left(1-\beta \left(1-\theta \right)\left(1-s\right)\right)}^{n}+\beta {\left(1-\left(\beta +\theta -\beta \theta \right)\left(1-s\right)\right)}^{n} \end{array}$$

The above equation implies that the term $\sum_{i=1}^{n}U_{i}$ has a distribution of:

$$U_{1}+U_{2}+\ldots +U_{n}=\left\{\begin{array}{cc} Bin(n,\beta (1-\theta )) & w.p\;1-\beta \\ Bin(n,\beta +\theta -\beta \theta ) & w.p\;\beta \end{array}\right.$$

Then the probability generating function (PGF) of $\left\{X_{t}\right\}$ is:

$$\phi_{X_{n}}\left(s\right)=\left[\left(1-\beta \right)\cdot \phi_{\varepsilon }\left(1-\beta \left(1-\theta \right)\left(1-s\right)\right)+\beta \cdot \phi_{\varepsilon }\left(1-\left(\beta +\theta -\beta \theta \right)\left(1-s\right)\right)\right]\cdot \phi_{\varepsilon }\left(s\right)$$

$\phi_{\varepsilon }\left(s\right)$ is the probability generating function of the $\varepsilon_{t}$. Given the distribution of $\varepsilon_{t}$, the explicit expression of the probability generating function can be derived. Suppose Poisson distribution of $\varepsilon_{t}$, then

$$\phi_{X_{n}}\left(s\right)=\left[\left(1-\beta \right)\cdot e^{-\lambda \beta (1-\theta )(1-s)}+\beta e^{-\lambda (\beta +\theta -\beta \theta )(1-s)}\right]\cdot e^{\left(\lambda (s-1)\right)}$$

The probability generating function is defined by the probabilities. The uniqueness of a power series expansion implies that the probability generating function in turn defines probabilities.

Therefore, we can derive the probability of $\left\{X_{t}\right\}$. For $X_{t}=j$, the probability mass function of $X_{t}$ is:

$$P\left(X_{t}=j\right)={\left[\frac{1}{j!}\frac{d^{j}\phi \left(s\right)}{ds^{j}}\right]}_{s=0}$$

The bivariate probability generating function of $\left\{X_{t}\right\}$ is $\Phi_{X_{t},X_{t-1}}\left(s_{1},s_{2}\right)$. Thus, deriving the explicit expression of bivariate probability generating function with Poisson innovation is easy for DCINMA(1) process.

$$\begin{array}{cc} E(s_{1}^{X_{t}}s_{2}^{X_{t-1}}) & =E(s_{1}^{\beta \circ \varepsilon_{t-1}+\varepsilon_{t}}\cdot s_{2}^{\beta \circ \varepsilon_{t-2}+\varepsilon_{t-1}})\\ \; & =E(s_{1}^{\beta \circ \varepsilon_{t-1}}\cdot s_{1}^{\varepsilon_{t}}\cdot s_{2}^{\beta \circ \varepsilon_{t-2}}\cdot s_{2}^{\varepsilon_{t-1}})\\ \; & =E\left(s_{1}^{\varepsilon_{t}}\right)\cdot E\left(s_{1}^{\beta \circ \varepsilon_{t-2}}\right)\cdot E\left(s_{1}^{\beta \circ \varepsilon_{t-2}}\cdot s_{2}^{\varepsilon_{t-1}}\right) \end{array}$$

Given the Poisson distribution of the innovation term, we can obtain:

$$E\left(s_{1}^{\varepsilon_{t}}\right)=e^{\lambda (s_{1}-1)},E\left(s_{2}^{\beta \circ \varepsilon_{t-1}}\right)=\left(1-\beta \right)\cdot e^{-\lambda \beta \left(1-\theta \right)\left(1-s_{2}\right)}+\beta \cdot e^{-\lambda \left(\beta +\theta -\beta \theta \right)\left(1-s_{2}\right)}$$

$$E\left(s_{1}^{\beta \circ \varepsilon_{t-1}}\cdot s_{2}^{\varepsilon_{t-1}}\right)=\left(1-\beta \right)\cdot e^{\lambda s_{2}\left[1-\beta \left(1-\theta \right)\left(1-s_{2}\right)\right]}+\beta \cdot e^{\lambda s_{2}\left[\left(1-\beta -\theta -\beta \theta \right)\left(1-s_{1}\right)\right]}.$$

### 2.4. Compare with the INMA(q) Model

The mean and the covariance of the *q*-th order integer-valued moving average model has the same expression, and the variance of the INMA(*q*) process is:

$$V_{inma}=\sigma_{\varepsilon }^{2}+\sum_{i=1}^{q}\left[\sigma_{\varepsilon }^{2}\beta_{i}+\mu_{\varepsilon }\beta_{i}\left(1-\beta_{i}\right)\right]$$

From Theorem 1, the variance of the DCINMA process presents a more complicated expression than the INMA(*q*) process due to the correlation between the counting series of parameter θ. For the Poisson innovation, the overdispersion index of the INMA and DCINMA model is as follows:

$$I_{inma}=1,I_{dinma}=\frac{1+\beta +\beta \lambda \theta^{2}\left(1-\beta \right)}{1+\beta }$$

Since the value of λ, β and θ are all non-negative, the term $1+\beta \lambda \theta^{2}\left(1-\beta \right)>1$. When two models (INMA(1) and DCINMA(1)) share the same λ and β, the θ will determine whether there is dependence between counting series (θ≠0). Thus, if we want to test θ=0, it is equivalent to evaluating whether the model is overdispersed. If the value of θ is 0, the model degenerates to INMA model.

### 2.5. Compare the Entropy with INMA(1) Model

Entropy is an important concept in physics, but it can also be applied to other disciplines, including cosmology and economics. Entropy is a measure of the randomness or disorder of a system. In our case, entropy can be seen as a dispersion measure for the model. Thus, we evaluate the model from the perspective of entropy. The definition of Shannon entropy as follows:

$$H\left(Y\right)=-\sum_{i=1}^{n}p\left(y_{i}\right)\cdot lnp\left(y_{i}\right)$$

where *Y* is a discrete random variable with probability mass function taking values on $y_{1},\ldots ,y_{n}$. We denote the $\phi_{dinma}\left(s\right)$ and $\phi_{inma}\left(s\right)$ as probability generating functions of the DCINMA(1) and INMA(1) process, respectively. Suppose the same innovation term for the two models follows the Poisson distribution. We can rewrite the probability generating function of them as:

$$\begin{array}{cc} \phi_{dinma}\left(s\right) & =\left(1-\beta \right)\cdot e^{\left(s-1)\cdot [\lambda \beta (1-\theta )+\lambda \right]}+\beta \cdot e^{\left(s-1)\cdot [\lambda (\beta +\theta -\beta \theta +\lambda )\right]}\\ \phi_{inma}\left(s\right) & =e^{\left(s-1)\cdot (\lambda \beta +\lambda \right)} \end{array}$$

Thus, we can conclude the distribution of DCINMA(1) and INMA(1) process based on the definition of the probability generating function. $X_{t}^{inma(1)}$ and $X_{t}^{dinma(1)}$ denote the sample from INMA(1) model and DCINMA(1) model.

$$\begin{array}{cc} X_{t}^{inma(1)} & ∼Poi(\lambda \beta +\lambda )\\ X_{t}^{dinma(1)} & ∼\left\{\begin{array}{cc} Poi(\lambda \beta (1-\theta )+\lambda ) & w.p\;1-\beta \\ Poi(\lambda (\beta +\theta -\beta \theta )+\lambda ) & w.p\;\beta \end{array}\right. \end{array}$$

The Shannon entropy for both models can be derived as follows:

$$\begin{array}{cc} H(X_{t}^{inma(1)}) & =\left(\lambda \beta +\lambda \right)\left[1-log\left(\left(\lambda \beta +\lambda \right)\right)\right]+e^{\left(\lambda \beta +\lambda \right)}\cdot \sum_{k=0}^{\infty }\frac{{\left(\lambda \beta +\lambda \right)}^{k}log\left(k!\right)}{k!}\\ H(X_{t}^{dinma(1)}) & =(1-\beta )\cdot \{(\lambda \beta (1-\theta )+\lambda )[1-log((\lambda \beta (1-\theta )+\lambda ))]\\ \; & +e^{\left(\lambda \beta (1-\theta )+\lambda \right)}\cdot \sum_{k=0}^{\infty }\frac{{\left(\lambda \beta \left(1-\theta \right)+\lambda \right)}^{k}log\left(k!\right)}{k!}\}\\ \; & +\beta \cdot \{(\lambda (\beta +\theta -\beta \theta )+\lambda )[1-log((\lambda (\beta +\theta -\beta \theta )+\lambda ))]\\ \; & +e^{\left(\lambda (\beta +\theta -\beta \theta )+\lambda \right)}\cdot \sum_{k=0}^{\infty }\frac{{\left(\lambda \left(\beta +\theta -\beta \theta \right)+\lambda \right)}^{k}log\left(k!\right)}{k!}\} \end{array}$$

The expression of entropy for the DCINMA(1) model is more complicated than the INMA(1) model due to the additional parameter θ.

## 3. Parameter Estimation

The estimation of the INMA model is complicated. Brännäs and Hall [27] discussed the Yule–Walker estimator, generalized moment method (GMM) based on the probability generating function (PGF) function, and the conditional least square method. Here, we did not attempt to use maximum likelihood estimation, which requires density functions that are generally not easily obtained in the INMA model, especially for this dependent situation. The results of generalized moments method-based probability generating function (PGF) function estimator are highly correlated with the values of $z_{1}$ and $z_{2}$ in $\Phi_{k}\left(z_{1},z_{2}\right)$, which are not stable. On the other hand, for the conditional least square method, the number of estimation equations are less than the number of parameters. This means that there is an additional parameter θ to be estimated.

Therefore, we derive the Yule–Walker estimator to obtain the unknown parameters for the Poisson DCINMA(*q*) model. We denote the following symbols: $\mu_{X}$ is the sample mean of $\left\{X_{t}\right\}$, $\mu_{\varepsilon }$ is the mean of innovation term $\varepsilon_{t}$. $\gamma_{0}$ is the sample variance of $\left\{X_{t}\right\}$, $\gamma_{1},\gamma_{2},\ldots ,\gamma_{q}$ are the sample covariance of 1-*th*, 2-*th*,…, *q*-*th* order. The $\beta_{0}$ for the equations below is 1.

Then, the unknown parameters in the DCINMA(*q*) model can be solved by equations through the sample moments function, which is as follows:

$$\begin{array}{c} \left\{\begin{array}{c} \mu_{X}=\mu_{\varepsilon }\left(1+\beta_{1}+\beta_{2}+\ldots +\beta_{q}\right)\\ \gamma_{0}=\sigma_{\varepsilon }^{2}+\sum_{i=1}^{q}\left[\mu_{\varepsilon }\beta_{i}+\mu_{\varepsilon }^{2}\theta^{2}\beta_{i}\left(1-\beta_{i}\right)-\mu_{\varepsilon }\left(\theta^{2}\beta_{i}-\theta^{2}\beta_{i}^{2}+\beta_{i}^{2}\right)+\beta_{i}^{2}\sigma_{\varepsilon }^{2}\right]\\ \gamma_{1}=\lambda \left(\beta_{0}\beta_{1}+\beta_{1}\beta_{2}+\ldots +\beta_{q-1}\beta_{q}\right)\\ \gamma_{2}=\lambda \left(\beta_{0}\beta_{2}+\beta_{1}\beta_{3}+\ldots +\beta_{q-2}\beta_{q}\right)\\ \;\;\;\;\;\;\;\;\vdots \\ \gamma_{q-1}=\lambda \left(\beta_{0}\beta_{q-1}+\beta_{1}\beta_{q}\right)\\ \gamma_{q}=\lambda \left(\beta_{0}\beta_{q}\right) \end{array}\right. \end{array}$$

## 4. Simulation Study

### 4.1. Estimation of the Model Parameters

In this section, we present some simulation results to show the performance of the estimator using different sample sizes. (n=100,300,700,1000). We focused on the Poisson DCINMA(1) model. The three group parameter values considered in this model are as follows:

$$\begin{array}{c} \mathrm{Model}\;A:(\lambda =1,\;\theta =0.6,\;\beta =0.2)\\ \mathrm{Model}\;B:(\lambda =4,\;\theta =0.7,\;\beta =0.1)\\ \mathrm{Model}\;C:(\lambda =5,\;\theta =0.5,\;\beta =0.1) \end{array}$$

We used the above parameter groups to generate the data and applied Yule–Walker method, then computed the bias and standard error based on 10,000 replications for each parameter group. The estimation results and their performance are reported in Table 1.

As shown in Table 1, we obtained nearly convergent estimators in all cases. In all cases, as the sample size increased, the bias decreased.

### 4.2. Testing for Dependence between Counting Series

From Section 2.5, when the two processes have the same λ and β, the DCINMA model always presents overdispersion due to θ. We tested whether the value of θ is 0, which is equivalent to assessing whether the DCINMA process presents overdispersion. Aleksandrov and Weiß [28] proposed the diagnostic test for the INMA process. In this section, only the overdispersion test was applied. Under the null hypothesis $H_{0}:\theta =0$ (the process does not present overdispersion), the distribution of index dispersion has the following form:

$${\hat{I}}_{disp}\overset{d}{⟶}N\left(1-\frac{1}{T}\frac{1+3\beta }{1+\beta },\frac{1}{T}\left(2+4{\left(\frac{\beta }{1+\beta }\right)}^{2}\right)\right)$$

We then analyzed the simulation results to assess the performance of the overdispersion test. The nominal level α=0.05 was employed, for sample sizes, n=100,150,250. We did 1000 replications to calculate the size and power of the test. The following parameter groups were considered:

$$\begin{array}{c} \mathrm{Model}\;D:(\lambda =3,\;\theta =0.4,\;\beta =0.5)\\ \mathrm{Model}\;E:(\lambda =5,\;\theta =0.4,\;\beta =0.3)\\ \mathrm{Model}\;F:(\lambda =6,\;\theta =0.3,\;\beta =0.4)\\ \mathrm{Model}\;G:(\lambda =7,\;\theta =0.7,\;\beta =0.8) \end{array}$$

From Table 2, given the same values for λ and β, under the null hypothesis, θ=0, the size of ${\hat{I}}_{disp}$ are close to nominal level. Under the alternative situation, θ≠0, for all cases, as *n* increase the power quickly increases to 1.

## 5. Real Data Example

We then applied the proposed model to a real dataset. Unlike the integer-valued autoregressive model, since the density function of this DCINMA process is hard to obtain, the Akaike information criterion (AIC) is difficult to use in measuring the fitness of such an INMA-type model. To assess the performance of the model, we adapted the parametric resampling method by Jung et al. [29].

We used crime data available from the Forecasting Principles site. The data consist of 144 observations of monthly larceny counts for the City of Pittsburgh from January of 1990 to December of 2001. It would be highly improbable for criminals to remain in the same place for a long time. They probably would flee in various directions to commit offenses, so that the INMA-type model is appropriate in this case.

**Figure 1:**
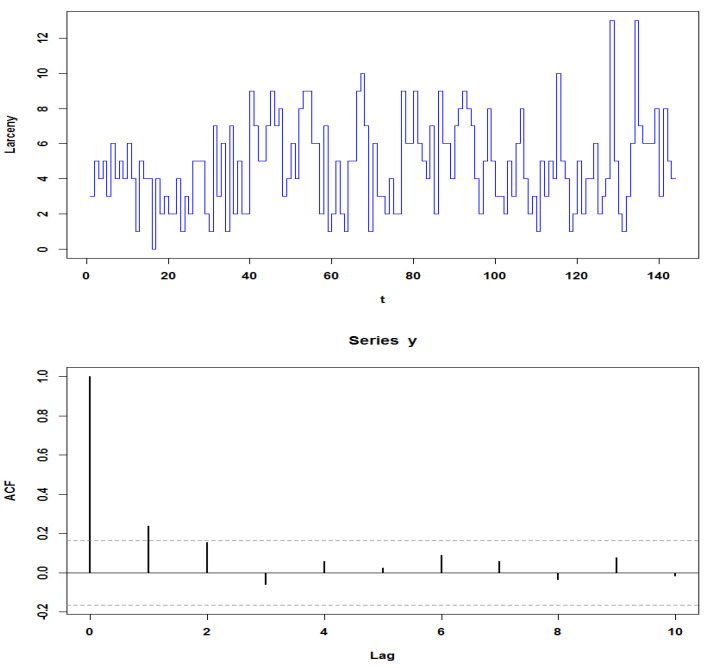
Time series plot (top figure) and autocorrelation function (below figure panel) for actual Larceny dataset.

The calculated of sample mean (4.73) and variance (6.40) suggest that the dataset is overdispersed. In addition, the sample autocorrelation function (ACF) in Figure 1 shows that the order of the model should be set to one. We fitted the data set into the DCINMA(1) model and INMA(1) model to evaluate the model performance for overdispersed data. The estimation results for the two models are presented in Table 3.

The parametric resampling method can be performed in several steps:

**Step 1:** Generate samples with length equal to the original dataset from the fitted model for *R* times.

**Step 2:** Compute the autocorrelation function (ACF) for each sample series. Then derive the empirical sample autocorrelation function (ACF) for the different lag orders.

**Step 3:** Using the results from **Step 2**, compute the 100(1−α/2) and 100(α/2) quantiles.

**Figure 2:**
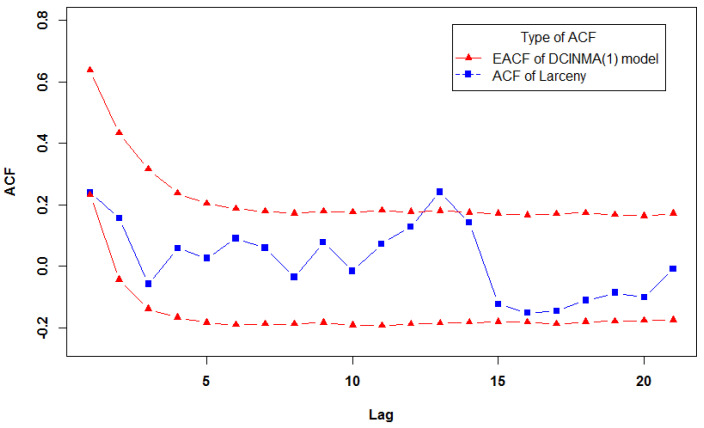
Acceptance envelope of the DCINMA(1) model for the autocorrelation function for the larceny dataset.

**Figure 3:**
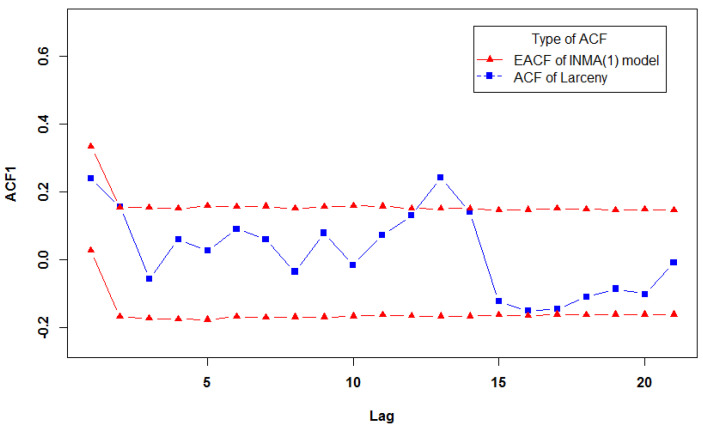
Acceptance envelope of the INMA(1) model for the autocorrelation function for the larceny dataset.

For the given example, *R* was set to 5000, and α was set to 0.05. We plotted the acceptance envelope for DCINMA(1) model and INMA(1) model, and the results are shown in Figure 2 and Figure 3. The sample autocorrelations for the real dataset lie within the acceptance envelopes of the DCINMA(1) model except for only one point, while the important second order of the autocorrelation function (ACF) exceed the upper bound of the acceptance envelopes of the INMA(1) model. It is clear that the acceptance envelopes of the DCINMA(1) model performs better than the acceptance envelopes of the INMA(1) model. Thus, the proposed model is suitable for this dataset, adequately representing the autocorrelation for real dataset.

## 6. Conclusions

In this paper, we constructed a new integer-valued moving average model with dependent counting series. The statistical properties of the proposed model were discussed and evaluated. The parameter estimation of the proposed model is based on the Yule–Walker method. The new model presents overdispersion due to the dependence parameter θ, which means the dependence between counting series can be verified by an overdispersion test. Numerical simulation results were used to evaluate the performance of the estimation and overdispersion test.

Future extensions for this study are as follows. First, we only focused on the stationary DCINMA(1) model in this paper. However, cases with non-stationary series are more common. The switched system is an important model in studying hybrid systems, particularly from the perspective of control science and engineering. Refer to ([30,31,32]) for more detailed discussion. The mechanism conducts the transformation between different subsystems, providing an approach to generalize the DCINMA process into a non-stationary case. A function can be introduced to control the change for different parameter values of the stationary case, which characterize non-stationary in series. Second, the parameter θ in our model provides a probability for overdispersion. In a switched system, the switching signal is a piecewise constant function, depending on time, external signal, output, and its own past value. Thus, the weighted switching signal function with weights θ and (1−θ) can be considered, where the weight θ gives the probability for switching to different subsystems.

## Figures and Tables

**Table 1:** Some numerical results of the estimates for true values of the parameters λ, θ and β.

Sample Size	$\hat{\lambda }$	$\hat{\theta }$	$\hat{\beta }$
(a) True values: λ = 1, θ = 0.6, β = 0.2			
100	0.9875	0.5999	0.2641
Bias	0.0124	0.0001	−0.0641
Standard Error	0.1755	0.2065	0.1705
300	0.9858	0.6192	0.2627
Bias	0.0141	−0.0192	−0.0627
Standard Error	0.1082	0.2049	0.1085
700	0.9831	0.6489	0.2663
Bias	0.0168	−0.0489	−0.0663
Standard Error	0.0699	0.1922	0.0717
1000	0.9817	0.6718	0.2684
Bias	0.0182	−0.0718	−0.0684
Standard Error	0.0597	0.1829	0.0600
(b) True values: λ = 4, θ = 0.7, β = 0.1			
100	3.7816	0.6173	0.1690
Bias	0.2183	0.0826	−0.0690
Standard Error	0.5296	0.2031	0.1353
300	3.8872	0.6611	0.1394
Bias	0.1127	0.0388	−0.0394
Standard Error	0.3364	0.1767	0.0855
700	3.9123	0.7208	0.1324
Bias	0.0876	−0.0208	−0.0324
Standard Error	0.2337	0.1353	0.0599
1000	3.9144	0.7430	0.1322
Bias	0.0855	−0.0430	−0.0322
Standard Error	0.2012	0.1142	0.0514
(c) True values: λ = 5, θ = 0.5, β = 0.1			
100	4.7799	0.5709	0.1556
Bias	0.2200	−0.0709	−0.0556
Standard Error	0.5777	0.2059	0.1142
300	4.9129	0.5703	0.1279
Bias	0.0870	−0.0703	−0.0279
Standard Error	0.3715	0.1852	0.0730
700	4.9343	0.5688	0.1243
Bias	0.0656	−0.0688	−0.0243
Standard Error	0.2615	0.1529	0.0520
1000	4.9340	0.5738	0.1245
Bias	0.0659	−0.0738	−0.0245
Standard Error	0.2211	0.1330	0.0442

**Table 2:** Size and power of DCINMA(1) and INMA(1) model.

λ	β	θ	*n*		
			100	250	500
3	0.4	0	0.054	0.057	0.058
		0.5	0.431	0.877	0.937
5	0.4	0	0.053	0.057	0.047
		0.3	0.447	0.824	0.901
6	0.3	0	0.052	0.055	0.047
		0.4	0.63	0.8	0.99
7	0.7	0	0.052	0.046	0.053
		0.8	0.998	1	1

**Table 3:** Estimation results of DCINMA(1) and INMA(1) model.

	λ	β	θ
DCINMA(1)	3.21	0.47	0.80
INMA(1)	3.82	0.24	0

## Data Availability

The data used to support the findings of this study are included within the article.

## Appendix A

**Proof of Theorem** **1.** we give the detailed derivation process for 1-th order, the *q*-th order can be derived in the same manner. term (i) and (ii) of the Theorem 1 are easy to verify, so we focus on the term (iii).

$$\begin{array}{cc} Var(X_{t}) & =Var\left(\beta \circ \varepsilon_{t-1}\right)+Var\left(\varepsilon_{t}\right)\\ \; & =V\left[E\left[\beta \circ \varepsilon_{t-1}|\varepsilon_{t-1}\right]\right]+E\left[V\left[\beta \circ \varepsilon_{t-1}|\varepsilon_{t-1}\right]\right]+Var\left(\varepsilon_{t}\right) \end{array}$$

Then, we focus on the term $V[\beta \circ \varepsilon_{t-1}|\varepsilon_{t-1}]$.

$$\begin{array}{cc} V[\beta \circ \varepsilon_{t-1}|\varepsilon_{t-1}] & =E\left[{\left(\sum_{i=1}^{\varepsilon_{t-1}}U_{i}\right)}^{2}|\varepsilon_{t-1}\right]-{\left[E\left[\sum_{i=1}^{\varepsilon_{t-1}}U_{i}|\varepsilon_{t-1}\right]\right]}^{2}\\ \; & =\varepsilon_{t-1}\cdot E\left[U_{1}^{2}\right]+\left(\varepsilon_{t-1}-1\right)\cdot \varepsilon_{t-1}\cdot E\left[U_{1}\cdot U_{2}\right]-{\left[\varepsilon_{t-1}\cdot \beta \right]}^{2}\\ \; & =\varepsilon_{t-1}\cdot \left(\beta \cdot \left(1-\beta \right)+\beta^{2}\right)+\varepsilon_{t-1}\cdot \left(\varepsilon_{t-1}-1\right)\cdot \left(\theta^{2}\cdot \beta \cdot \left(1-\beta \right)+\beta^{2}\right)\\ \; & -\beta^{2}\cdot \varepsilon_{t-1}^{2} \end{array}$$

$$\begin{array}{cc} E[V\left[\beta \circ \varepsilon_{t-1}|\varepsilon_{t-1}\right]] & =\left(\beta \cdot \left(1-\beta \right)+\beta^{2}\right)\cdot E\left[\varepsilon_{t-1}\right]-E\left[\beta^{2}\cdot \varepsilon_{t-1}^{2}\right]\\ \; & +E\left[\varepsilon_{t-1}\cdot \left(\varepsilon_{t-1}-1\right)\right]\cdot \left(\theta^{2}\cdot \beta \cdot \left(1-\beta \right)+\beta^{2}\right)\\ \; & =\lambda \cdot \left(\beta \cdot \left(1-\beta \right)-\beta^{2}\right)-\beta^{2}\cdot \left(\lambda +\lambda^{2}\right)\\ \; & +\left(\lambda +\lambda^{2}-\lambda \right)\cdot \left(\theta^{2}\cdot \beta \cdot \left(1-\beta \right)+\beta^{2}\right)\\ \; & =\lambda^{2}\cdot \beta \cdot \left(1-\beta \right)\cdot \theta^{2}+\beta \cdot \lambda -\beta^{2}\cdot \lambda \end{array}$$

$$\begin{array}{cc} Var(X_{t}) & =V\left[\beta \cdot \varepsilon_{t-1}\right]\lambda^{2}\cdot \beta \cdot \left(1-\beta \right)\cdot \theta^{2}+\beta \cdot \lambda -\beta^{2}\cdot \lambda +\lambda \\ \; & =\beta \cdot \lambda +\lambda^{2}\cdot \beta \cdot \left(1-\beta \right)\cdot \theta^{2}+\lambda \end{array}$$

 ☐

**Proof of Theorem** **3.** let $Y_{t}=X_{t}-\mu_{X}$, $\left\{X_{t}\right\}$ is the process defined in Section 2. To prove the $Y_{t}$ is ergodic in autocovariance function, it is sufficient to show that:

$$\begin{array}{c} lim_{T\to \infty }\frac{1}{T}\sum_{l=0}^{T}\left\{E\left(Y_{t}Y_{t+v}Y_{t+l}Y_{t+v+l}\right)-R^{2}\left(v\right)\right\}=0 \end{array}$$

It is obvious $E(Y_{t}^{i})<\infty$. For i=1,2,3,4, let $E\left(Y_{t}^{2}\right)=c_{2},E\left(Y_{t}^{4}\right)=c_{4}$.
**The case v=0:**

$$\begin{array}{c} E\left(Y_{t}Y_{t+v}Y_{t+l}Y_{t+v+l}\right)=E\left(Y_{t}^{2}Y_{t+l}^{2}\right) \end{array}$$

If l=1,

$$\begin{array}{c} E\left(Y_{t}Y_{t+v}Y_{t+l}Y_{t+v+l}\right)=E\left(Y_{t}^{2}Y_{t+l}^{2}\right)\leq \left(E(\right|Y_{t}^{2}{|}^{2}|Y_{t+l}^{2}{|}^{2}{))}^{1/2}=c_{4} \end{array}$$

If l>1, $Y_{t}$ and $Y_{t+l}$ are irrelevant, then

$$\begin{array}{c} E\left(Y_{t}Y_{t+v}Y_{t+l}Y_{t+v+l}\right)=E\left(Y_{t}^{2}Y_{t+l}^{2}\right)=c_{2}^{2}=\gamma_{Y}^{2}\left(0\right)\\ \frac{1}{T}\sum_{l=0}^{T}\left\{E\left(Y_{t}Y_{t+v}Y_{t+l}Y_{t+v+l}\right)-R^{2}\left(v\right)\right\}\leq \frac{1}{T}\left\{c_{4}-\gamma_{Y}^{2}\left(0\right)\right\}\to 0 \end{array}$$

**The case v≥1:** If l≤v+1:

$$\begin{array}{c} E\left(Y_{t}Y_{t+v}Y_{t+l}Y_{t+v+l}\right)\leq \left[E(\right|Y_{t}Y_{t+v}{|}^{2}|Y_{t+l}Y_{t+l+v}{|}^{2}{]}^{\frac{1}{2}}\leq \left[E(\right|Y_{t}{|}^{4}|Y_{t+v}{|}^{4}|Y_{t+l}{|}^{4}|Y_{t+l+v}{{|}^{4}]}^{\frac{1}{4}}=c_{4} \end{array}$$

If l>v+1, $Y_{t}Y_{t+l}$ and $Y_{t+l}Y_{t+l+v}$ are irrelevant, then

$$\begin{array}{c} E\left(Y_{t}Y_{t+v}Y_{t+l}Y_{t+v+l}\right)=E\left(Y_{t}Y_{t+v}\right)E\left(Y_{t+l}Y_{t+l+v}\right)=R^{2}\left(v\right)\\ \frac{1}{T}\sum_{l=0}^{T}\left\{E\left(Y_{t}Y_{t+v}Y_{t+l}Y_{t+v+l}\right)-R_{v}^{2}\right\}\leq \frac{1}{T}\left\{\left(c_{4}-R^{2}\left(v\right)\right)\left(v+1\right)\right\}\to 0 \end{array}$$

We can obtain:

$$\begin{array}{c} lim_{T\to \infty }E\left[{\left({\hat{R}}_{n}^{2}\left(v\right)-R^{2}\left(v\right)\right)}^{2}\right]=0 \end{array}$$

Which implies:

$$\begin{array}{c} {\hat{R}}^{2}\left(v\right)\overset{p}{\to }R^{2}\left(v\right) \end{array}$$

The above equation is holding for variable $Y_{t}$:

$$Y_{t}=X_{t}-\mu_{X},{\hat{R}}_{Y}^{2}\left(v\right)\overset{p}{\to }R_{Y}^{2}\left(v\right)$$

We need to prove that

$$\begin{array}{c} P(|{\hat{R}}_{X}^{2}\left(v\right)-R_{X}^{2}\left(v\right)|\geq \epsilon )\to 0 \end{array}$$

Because of $R_{X}^{2}\left(v\right)=R_{Y}^{2}\left(v\right)$, rewriting the above equation as

$$\begin{array}{cc} P(|{\hat{R}}_{X}^{2}\left(v\right)-R_{X}^{2}\left(v\right)|\geq \epsilon )\; & =P(|{\hat{R}}_{X}^{2}\left(v\right)-{\hat{R}}_{Y}^{2}\left(v\right)+{\hat{R}}_{Y}^{2}\left(v\right)-R_{X}^{2}\left(v\right)|\geq \epsilon )\\ \; & \leq P(|{\hat{R}}_{X}^{2}\left(v\right)-{\hat{R}}_{Y}^{2}\left(v\right)|\geq \epsilon /2)+P(|{\hat{R}}_{Y}^{2}\left(v\right)-R_{Y}^{2}\left(v\right)\left|\geq \epsilon /2\right) \end{array}$$

${\hat{R}}_{Y}^{2}\left(v\right)$ and ${\hat{R}}_{X}^{2}\left(v\right)$ expressing as

$$\begin{array}{c} {\hat{R}}_{X}^{2}\left(v\right)=\frac{1}{T}\sum_{t=1}^{T-k}\left(X_{t+k}-\bar{X}\right)\left(X_{t}-\bar{X}\right),{\hat{R}}_{Y}^{2}\left(v\right)=\frac{1}{T}\sum_{t=1}^{T-k}Y_{t+k}Y_{t}=\frac{1}{T}\sum_{t=1}^{T-k}\left(X_{t+k}-\mu_{X}\right)\left(X_{t}-\mu_{X}\right) \end{array}$$

Then the following expression can be derived

$$\begin{array}{cc} P(|{\hat{R}}_{X}^{2}\left(v\right)-{\hat{R}}_{Y}^{2}\left(v\right)|\geq \epsilon /2)\; & =P(\left({\bar{X}}^{2}-\mu_{X}^{2}\right)+\left(\mu_{X}-\bar{X}\right)\left({\bar{X}}_{t+k}+{\bar{X}}_{T}\right)\geq \epsilon /2)\\ \; & \leq P\left({\bar{X}}^{2}-\mu_{X}^{2}\geq \varepsilon /4\right)+P(\left(\mu_{X}-\bar{X}\right)\left({\bar{X}}_{t+k}+{\bar{X}}_{T}\right)\\ \; & \geq \varepsilon /4) \end{array}$$

Since $\bar{X}\overset{p}{\to }\mu_{X}$, by Slutsky’s theorem

$$\begin{array}{c} {\bar{X}}^{2}-\mu_{X}^{2}\overset{p}{\to }0,\left(\mu_{X}-\bar{X}\right)\left({\bar{X}}_{t+k}+{\bar{X}}_{T}\right)\overset{p}{\to }0 \end{array}$$

Consequently,

$$\begin{array}{c} P(|{\hat{R}}_{X}^{2}\left(v\right)-R_{X}^{2}\left(v\right)|\geq \epsilon )\to 0,T\to \infty \end{array}$$

 ☐
